# Analysis of Pregnancy Complications and Epigenetic Gestational Age of Newborns

**DOI:** 10.1001/jamanetworkopen.2023.0672

**Published:** 2023-02-24

**Authors:** Christine Ladd-Acosta, Elizabeth Vang, Emily S. Barrett, Catherine M. Bulka, Nicole R. Bush, Andres Cardenas, Dana Dabelea, Anne L. Dunlop, Rebecca C. Fry, Xingyu Gao, Jaclyn M. Goodrich, Julie Herbstman, Marie-France Hivert, Linda G. Kahn, Margaret R. Karagas, Elizabeth M. Kennedy, Anna K. Knight, Sahra Mohazzab-Hosseinian, Andréanne Morin, Zhongzheng Niu, T. Michael O’Shea, Meredith Palmore, Douglas Ruden, Rebecca J. Schmidt, Alicia K. Smith, Ashley Song, Eliot R. Spindel, Leonardo Trasande, Heather Volk, Daniel J. Weisenberger, Carrie V. Breton

**Affiliations:** 1 Department of Epidemiology, Johns Hopkins Bloomberg School of Public Health, Baltimore, Maryland; 2 Department of Population and Public Health Sciences, Keck School of Medicine, University of Southern California, Los Angeles; 3 Department of Biostatistics and Epidemiology, Environmental and Occupational Health Sciences Institute, Rutgers School of Public Health, Piscataway, New Jersey; 4 Department of Environmental Sciences and Engineering, Gillings School of Global Public Health, University of North Carolina, Chapel Hill; 5 Department of Psychiatry and Behavioral Sciences, University of California, San Francisco; 6 Department of Pediatrics, University of California, San Francisco; 7 Department of Epidemiology and Population Health, Stanford University, Stanford, California; 8 Lifecourse Epidemiology of Adiposity and Diabetes Center, University of Colorado Anschutz Medical Campus, Aurora; 9 Department of Gynecology and Obstetrics, Emory University School of Medicine, Atlanta, Georgia; 10 Department of Environmental Health Sciences, School of Public Health, University of Michigan, Ann Arbor; 11 Department of Environmental Health Sciences, Mailman School of Public Health, Columbia University, New York, New York; 12 Department of Population Medicine, Harvard Pilgrim Health Care Institute, Harvard Medical School, Boston, Massachusetts; 13 Department of Pediatrics, New York University Grossman School of Medicine, New York, New York; 14 Department of Population Health, New York University Grossman School of Medicine, New York, New York; 15 Department of Epidemiology, Geisel School of Medicine at Dartmouth, Hanover, New Hampshire; 16 Gangarosa Department of Environmental Health, Emory Rollins School of Public Health, Atlanta, Georgia; 17 Department of Human Genetics, University of Chicago, Chicago, Illinois; 18 Department of Pediatrics, University of North Carolina School of Medicine, Chapel Hill; 19 Department of Obstetrics and Gynecology, Wayne State University, Detroit, Michigan; 20 Division of Environmental and Occupational Health and Epidemiology, Department of Public Health Sciences and the MIND Institute, School of Medicine, University of California, Davis; 21 Department of Psychiatry and Behavioral Sciences, Emory University School of Medicine, Atlanta, Georgia; 22 Department of Mental Health, Johns Hopkins Bloomberg School of Public Health, Baltimore, Maryland; 23 Division of Neuroscience, Oregon National Primate Research Center, Oregon Health & Science University, Beaverton; 24 Department of Biochemistry and Molecular Medicine, University of Southern California, Los Angeles

## Abstract

**Importance:**

Preeclampsia, gestational hypertension, and gestational diabetes, the most common pregnancy complications, are associated with substantial morbidity and mortality in mothers and children. Little is known about the biological processes that link the occurrence of these pregnancy complications with adverse child outcomes; altered biological aging of the growing fetus up to birth is one molecular pathway of increasing interest.

**Objective:**

To evaluate whether exposure to each of these 3 pregnancy complications (gestational diabetes, gestational hypertension, and preeclampsia) is associated with accelerated or decelerated gestational biological age in children at birth.

**Design, Setting, and Participants:**

Children included in these analyses were born between 1998 and 2018 and spanned multiple geographic areas of the US. Pregnancy complication information was obtained from maternal self-report and/or medical record data. DNA methylation measures were obtained from blood biospecimens collected from offspring at birth. The study used data from the national Environmental Influences on Child Health Outcomes (ECHO) multisite cohort study collected and recorded as of the August 31, 2021, data lock date. Data analysis was performed from September 2021 to December 2022.

**Exposures:**

Three pregnancy conditions were examined: gestational hypertension, preeclampsia, and gestational diabetes.

**Main Outcomes and Measures:**

Accelerated or decelerated biological gestational age at birth, estimated using existing epigenetic gestational age clock algorithms.

**Results:**

A total of 1801 child participants (880 male [48.9%]; median [range] chronological gestational age at birth, 39 [30-43] weeks) from 12 ECHO cohorts met the analytic inclusion criteria. Reported races included Asian (49 participants [2.7%]), Black (390 participants [21.7%]), White (1026 participants [57.0%]), and other races (92 participants [5.1%]) (ie, American Indian or Alaska Native, Native Hawaiian or other Pacific Islander, multiple races, and other race not specified). In total, 524 participants (29.0%) reported Hispanic ethnicity. Maternal ages ranged from 16 to 45 years of age with a median of 29 in the analytic sample. A range of maternal education levels, from less than high school (260 participants [14.4%]) to Bachelor’s degree and above (629 participants [34.9%]), were reported. In adjusted regression models, prenatal exposure to maternal gestational diabetes (β, −0.423; 95% CI, −0.709 to −0.138) and preeclampsia (β, −0.513; 95% CI, −0.857 to −0.170), but not gestational hypertension (β, 0.003; 95% CI, −0.338 to 0.344), were associated with decelerated epigenetic aging among exposed neonates vs those who were unexposed. Modification of these associations, by sex, was observed with exposure to preeclampsia (β, −0.700; 95% CI, −1.189 to −0.210) and gestational diabetes (β, −0.636; 95% CI, −1.070 to −0.200), with associations observed among female but not male participants.

**Conclusions and Relevance:**

This US cohort study of neonate biological changes related to exposure to maternal pregnancy conditions found evidence that preeclampsia and gestational diabetes delay biological maturity, especially in female offspring.

## Key Points

**Question:**

Is exposure to gestational diabetes, gestational hypertension, or preeclampsia associated with biological gestational age, measured via epigenetic clocks, in newborns?

**Findings:**

In this national multisite cohort study of 1801 children, preeclampsia and gestational diabetes were significantly associated with decelerated gestational age in exposed offspring at birth vs unexposed offspring (ie, they were estimated to be biologically younger than their chronological gestational age), and these associations were more pronounced in female offspring. No associations were observed for gestational hypertension and accelerated or decelerated biological age.

**Meaning:**

These findings suggest that the epigenetic developmental aging pathway is an important biological pathway associated with 2 pregnancy conditions and may play a role in mediating the effects of these conditions on perinatal and child health outcomes, particularly among female offspring.

## Teaser

This cohort study evaluates whether exposure to each of 3 pregnancy complications (gestational diabetes, gestational hypertension, and preeclampsia) is associated with accelerated or decelerated gestational biological age in children at birth.

## Introduction

Maternal medical conditions arising during pregnancy are associated with increased risks of morbidity and mortality in both mothers and their offspring. Among the most serious of these conditions are gestational diabetes (GD), gestational hypertension (GHT), and preeclampsia (PE). The prevalence of each of these disorders has increased over the past several decades in the US. The prevalence of GD is estimated to have increased almost 20-fold in the interval between 1979 and 2010, and the prevalence of PE and GHT increased by 25% and 184%, respectively, between 1987 and 2004.^1^ These increasing rates of maternal pregnancy complications constitute a major public health concern given their impact on maternal and child health.

GD is associated with an increased risk of obstetric complications (eg, preterm delivery, macrosomia, and shoulder dystocia), neonatal disorders (eg, hypoglycemia, hyperbilirubinemia, and respiratory distress syndrome),^2^ and long-term effects on the offspring (eg, obesity, accelerated pubertal development, and youth-onset type 2 diabetes).^3,4^ In addition, after the index pregnancy, mothers with GD have a nearly 10-fold increased risk of developing type 2 diabetes compared with women with uncomplicated pregnancies,^5^ as well as increased risk of future cardiovascular disease even in the absence of type 2 diabetes.^6^ GHT and PE are associated with an increased risk of peripartum maternal organ failure and mortality,^7^ fetal growth restriction and its correlates (eg, neonatal hypoglycemia),^8^ and future cardiometabolic disease in both the mother and the offspring.^9,10,11,12,13,14^ Finally, both GD and GHT have been associated with attention-deficit/hyperactivity disorder and autism in offspring.^3,4,15^

Although rates of GD and PE have not been shown to differ by fetal sex, previous literature supports a sex-specific differential response to these conditions. For example, one study^16^ found that male but not female offspring born to women with GD were at greater risk of child overweight or obesity at ages 5 to 7 years. Another study^17^ found that low birth weight among offspring of women with PE led to increased body weight and blood pressure among male but not female offspring. These studies support the hypothesis that male and female fetuses may respond differently to pregnancy complications, including GD and PE, providing a rationale for conducting sex-specific analyses in this study.

Across an individual’s lifetime, DNA methylation of a subset of specific sites within the genome changes reliably over time and has been used to estimate epigenetic age. In adults, accelerated or decelerated epigenetic age (ie, being biologically older or younger than one’s chronological age, respectively), is associated with health outcomes, including mortality and longevity,^18,19,20,21^ cancer,^22,23^ and cardiovascular disease.^24^ Gestational epigenetic clocks, created on the basis of reliable DNA methylation patterns in cord blood^25,26^ or placenta,^27^ have been used to compute measures of biological gestational age acceleration (GAA) or gestational age deceleration. Accelerated epigenetic age of the neonate has been associated with prenatal factors, such as maternal prepregnancy overweight and obesity^28^ and vitamin levels,^29^ as well as infant outcomes, such as birth weight, birth length, and head circumference. GAA, which is based on umbilical cord blood and newborn blood spot DNA methylation, has also been associated with older maternal age and multiple prenatal characteristics.^30^ These observations led us to hypothesize that GD, GHT, and PE are associated with newborns’ gestational epigenetic age, providing a potential mechanism linking these pregnancy complications to offspring perinatal and postnatal health outcomes.

Given the dearth of data on gestational epigenetic clocks and the high prevalence of pregnancy complications, we leveraged the extensive data and resources of the Environmental Influences on Child Health Outcomes (ECHO) program^31^ to advance our understanding of the association between pregnancy complications and developmental epigenetic aging processes among offspring from multiple cohorts, with broad representation across the US. Given hypothesized sex-specific associations, we also assessed whether these associations differed between female and male offspring.

## Methods

### Study Population

The ECHO program is a multisite national program that aims to address critical questions about how the environment affects child health and development. ECHO brings together numerous observational cohorts from across the country, representing multiple life stages,^32^ and provides new and existing data as a collective resource to help answer research questions related to child health. To test our hypotheses, we applied 2 inclusion criteria to our analysis: participants (1) must have high-quality child DNA methylation data available on an Illumina platform that was collected from blood within 3 days of birth, and (2) must have data collected on the presence or absence of at least 1 of the 3 maternal pregnancy complications: GHT, GD, and PE. This cohort study used cohort data available through the ECHO data repository as of the August 31, 2021, data lock date. The study protocol was approved by the local or single ECHO institutional review board. The single institutional review board used for new ECHO data collection is WIRB Copernicus Group. Written informed consent or parent’s or guardian’s permission was obtained, along with child assent as appropriate, for ECHO-wide Cohort Data Collection Protocol participation and for participation in specific cohorts. This study follows the Strengthening the Reporting of Observational Studies in Epidemiology (STROBE) reporting guideline for cohort studies.

### Definition of Pregnancy Complications and Covariates

Participants were classified as having GHT, GD, or PE if they self-reported a doctor’s diagnosis of 1 of these conditions or if any of these conditions was indicated in their prenatal medical record per maternal medical record abstraction. Participants with preexisting chronic hypertension or diabetes were coded as “no” to having GHT or GD, respectively. Fewer than 10 participants had more than 1 pregnancy condition. Additional covariates, including infant sex, maternal age at delivery, self-reported race and ethnicity (Asian, American Indian or Alaska Native, Black, Hispanic, Native Hawaiian or other Pacific Islander, White, multiple races, and other race not specified), maternal education, prepregnancy body mass index (BMI; calculated as weight in kilograms divided by height in meters squared), and prenatal smoking were obtained from participant report on sociodemographic questionnaires or via medical records. Race and ethnicity were assessed in this study because we were concerned each could be associated with both changes in epigenetic age and pregnancy complications and was unlikely to be a mediator of pregnancy complication associations with epigenetic age. Chronological gestational age at birth, in weeks, was calculated from the last menstrual period or prenatal ultrasonography examination.

### Biospecimen Collection and DNA Methylation Data Measures

Cohorts collected cord blood or newborn blood spots through site-specific protocols and assayed DNA methylation using 1 of 3 Illumina BeadArray platforms: the Infinium HumanMethylation27 (HM27), Infinium HumanMethylation450 (HM450), or Infinium MethylationEPIC array, described in greater detail later. In total, 1 cohort provided HM27 data, 6 cohorts provided HM450 data, and 7 cohorts provided EPIC array data.

Raw .IDAT files from each cohort were imported into the minfi processing pipeline in R to calculate β values for each probe and sample. Beta values were calculated as [*M* / (*M* + *U*)], in which *M* and *U* refer to the mean methylated and unmethylated probe signal intensities, respectively. Measurements in which the fluorescent intensities were not statistically significantly above background signal (detection *P* > .05) were masked as missing. We applied sample-level filters to remove samples that had a low probe call rate (>1% probes with detection *P* > .05), discrepant estimated and reported sex, low overall array signal intensity, were duplicates, or failed bisulfite conversion or beadC quality criteria. A total of 1801 samples passed these quality control measures and had data on at least 1 pregnancy condition.

Next, we applied probe-level filters to filter data from probes that had detection *P* > .05, had beads that were cross-reactive to multiple locations in the genome, or contained known single-nucleotide variants.^33^ On the basis of these filters, 427 probes were removed from HM27, 54 057 probes were removed from HM450, and 184 393 probes were removed from the EPIC data set.

Background correction and normalization was performed on the filtered data sets for each Illumina DNA methylation platform using the noob function in minfi as first described by Triche et al.^34^ Finally, we performed additional sample filters on the noob-corrected data sets based on age discrepancies to achieve clean data sets for each platform. These clean data sets were used as input for gestational epigenetic age and cell composition estimation.

### Gestational Epigenetic Age Calculations

Given robust previous performance, the ability to use data from multiple Illumina array platforms, and the wide range of diverse gestational ages included, we chose to use the Knight gestational epigenetic clock here.^26^ The correlation coefficient in our analytic sample is 0.43 and had *P* < .001. Extrinsic age acceleration (EAA) was calculated using a linear regression model with chronological gestational age at birth (in completed weeks) as the independent variable and epigenetic age as the dependent variable. Extracted residuals from the linear regression model represent individuals’ EAA.

Intrinsic age acceleration (IAA) was calculated in 2 ways. First, a linear regression model was used with chronological gestational age at birth as the independent variable and epigenetic age as the dependent variable, adjusted for estimated cell proportions from different specimen source references. These were computed in the minfi Bioconductor package using the estimateCellCounts() function,^35^ with the compositeCellType argument set to “blood” for samples collected on dried blood spot cards and to “CordBlood” for cord blood samples. The cell types for peripheral blood spot samples included granulocytes, monocytes, natural killer cells, B cells, CD4 cells, and CD8 cells. For cord blood samples, the cell types accounted for include granulocytes, monocytes, natural killer cells, B cells, nucleated red blood cells, CD4 cells, and CD8 cells. The residuals extracted from the models represent individuals’ IAAs.

### Statistical Analysis

Data analysis was performed from September 2021 to December 2022. All data management and analysis were performed in R statistical software version 4.1.0 (R Project for Statistical Computing). Pregnancy complications and all covariates of interest, including maternal ethnicity, race, prepregnancy BMI, age, education level, and prenatal smoking, were summarized with descriptive statistics (frequency and percentage for categorical variables, and mean [SD] or median for continuous variables). Simple linear regression models were fitted with GD, GHT, and PE as exposures, and the 3 different measures of age acceleration (EAA, IAA, and sex-adjusted IAA) were independently tested as responses. To assess the impact of each covariate on the model, we performed a forward stepwise procedure, adding 1 covariate at a time. Point estimates, 95% CIs, and *P* values are reported. A 2-sided *P* < .05 was taken as evidence for a statistically significant association between each pregnancy complication and the corresponding age acceleration. Finally, we explored differences by child sex by repeating the aforementioned procedures among sex strata.

We adopted the multiple imputation method for covariates of interest with missing values. According to Rubin,^36^ the multiple imputation is a method for the estimation of incomplete data that accounts for the uncertainty around the true value under certain conditions. First, we performed multiple imputation by chained equations for each incomplete variable. For numeric variables (prepregnancy BMI), we used predictive mean matching; for dichotomous variables (ethnicity and smoking history), we used logistic regression; for the unordered categorical variable race, we used polytomous regression; and for the ordered categorical variable maternal educational level, we used a proportional odds model.

We checked the descriptive statistics of a random complete data set after multiple imputation, and no significant different was observed for all imputed covariates. After all variables were imputed, we fit the linear regression models to each of the 25 imputed data sets. Finally, the results were pooled using Rubin’s rules^36^ to account for uncertainty. In our imputation procedure, we took cohort differences into consideration to guarantee the missing at random assumption. We used the mice package for the whole imputation process that was developed by Stef van Buuren.^37^

## Results

In total 1801 participants, born between 1998 and 2018, from 12 cohorts met the inclusion criteria (Table 1 and eFigure in Supplement 1). A detailed description of the recruitment methods and key characteristics for each of the 12 cohorts that met our inclusion criteria is provided in eTable 1 in Supplement 1.

**Table 1.** Descriptive Statistics for Environmental Influences on Child Health Outcomes Participants Across 12 Cohorts (Birth Years 1998-2018)

Characteristic (N = 1801)	Gestational diabetes			Gestational hypertension			Preeclampsia		
	Participants, No. (%)		*P* value	Participants, No. (%)		*P* value	Participants, No. (%)		*P* value
	No (n = 1282)	Yes (n = 89)		No (n = 982)	Yes (n = 57)		No (n = 1401)	Yes (n = 57)	
Chronologic gestational age, median (range), wk	39.0 (30.0-43.0)	39.0 (34.0-42.0)	.01	39.0 (30.0-43.0)	39.0 (35.0-41.0)	.008	39.0 (31.0-43.0)	39.0 (34.0-43.0)	.003
Maternal age, mean (SD), y	29.40 (5.95)	31.50 (5.92)	.001	29.90 (6.08)	30.20 (6.73)	.78	28.10 (5.94)	27.30 (6.41)	.36
Child sex									
Male	639 (49.8)	51 (57.3)	.21	485 (49.4)	29 (50.9)	.94	680 (48.5)	28 (49.1)	>.99
Female	643 (50.2)	38 (42.7)		497 (50.6)	28 (49.1)		721 (51.5)	29 (50.9)	
Ethnicity									
Non-Hispanic	1045 (81.5)	59 (66.3)	.001	769 (78.3)	48 (84.2)	.49	931 (66.5)	26 (45.6)	.001
Hispanic	223 (17.4)	>25^a^		198 (20.2)	9 (15.8)		455 (32.5)	31 (54.4)	
Missing	14 (1.1)	<5^a^		15 (1.5)	0		15 (1.1)	0	
Race									
Asian	35 (2.7)	10 (11.2)	<.001	37 (3.8)	<5	.55	34 (2.4)	<5	.59
Black	206 (16.1)	11 (12.4)		115 (11.7)	10 (17.5)		343 (24.5)	8 (14.0)	
Missing	28 (2.2)	<10^a^		19 (1.9)	0		226 (16.1)	18 (31.6)	
Other^b^	74 (5.8)	<10^a^		56 (5.7)	<5		73 (5.2)	<5	
White	939 (73.2)	55 (61.8)		755 (76.9)	43 (75.4)		725 (51.7)	26 (45.6)	
Maternal prepregnancy body mass index, mean (SD)^c^	25.9 (6.4)	29.9 (7.7)	<.001	25.4 (6.1)	27.6 (7.4)	.04	26.2 (6.5)	29.9 (9.1)	.006
Missing	71 (5.5)	10 (11.2)		63 (6.4)	<5		121 (8.6)	7 (12.3)	
Maternal education level									
Less than high school	112 (8.7)	>10^a^	.48	101 (10.3)	<10	.80	230 (16.4)	>12^a^	.23
High school degree or equivalent	156 (12.2)	15 (16.9)		135 (13.7)	10 (17.5)		279 (19.9)	12 (21.1)	
Some college	248 (19.3)	20 (22.5)		206 (21.0)	10 (17.5)		265 (18.9)	16 (28.1)	
Bachelor’s degree and above	562 (43.8)	36 (40.4)		515 (52.4)	31 (54.4)		415 (29.6)	12 (21.1)	
Missing	204 (15.9)	<10^a^		25 (2.5)	<5^a^		212 (15.1)	<5^a^	
Maternal prenatal smoking									
No	871 (67.9)	65 (73.0)	.60	868 (88.4)	>52^a^	.10	1038 (74.1)	49 (86.0)	.22
Yes	64 (5.0)	<5^a^		63 (6.4)	0		86 (6.1)	<5^a^	
Missing	347 (27.1)	>19^a^		51 (5.2)	<5^a^		277 (19.8)	<10^a^	

^a^ It is an Environmental Influences on Child Health Outcomes requirement that table cells and figures that report data from fewer than 5 participants must be suppressed to protect participant confidentiality (ie, marked as <5), including summation of cells to prevent calculation of the exact sample size in cells with fewer than 5 participants.

^b^ Includes participants who reported any of the following: American Indian or Alaska Native, Native Hawaiian or other Pacific Islander, multiple races, and other race not specified.

^c^ Body mass index is calculated as weight in kilograms divided by height in meters squared.

The median (range) chronological gestational age at birth was 39 (30-43) weeks. Offspring male-to-female sex ratio was close to the expected 50:50 ratio (880 male [48.9%]). The majority of our analytic sample self-reported White race (1026 participants [57.0%]) and non-Hispanic ethnicity (1227 participants [71.0%]), but we also had representation of participants reporting Hispanic ethnicity (524 participants [29.0%]) and Asian (49 participants [2.7%]), Black (390 participants [21.7%]), and other (92 participants [5.1%]) races (ie, American Indian or Alaska Native, Native Hawaiian or other Pacific Islander, multiple races, and other race not specified). Notably, this is a larger proportion of individuals of Hispanic ethnicity than reported in the most recent US Census. Most of our participants had reported a maternal education level of a bachelor’s degree or higher (629 participants [34.9%]), but we had substantial representation of individuals in other education categories, including less than a high school degree (260 participants [14.4%]), a high school degree or equivalent (326 participants [18.0%]), and some college (364 participants [19.0%]). We checked the descriptive statistics of a random complete data set after multiple imputation, and no significant difference was observed for all imputed covariates. Demographic characteristics of our analytic sample of 1801 participants also did not differ substantively from those in the overall sample of 1962 participants (eTable 2 in Supplement 1).

**Figure 1.**
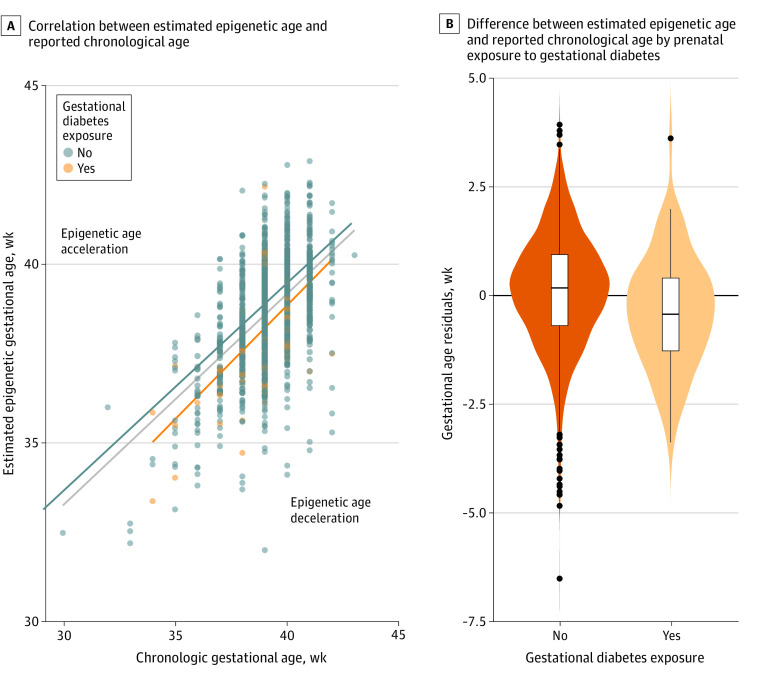
Association of Prenatal Exposure to Gestational Diabetes With Decelerated Epigenetic Gestational Age in Neonates A, Graph shows correlation between estimated epigenetic age and reported chronological age. Each data point represents a participant’s gestational age at birth according to exposure to gestational diabetes during development. Lines represent the average residual values among a group of participants with or without exposure and in the overall sample (dark blue line). B, Violin plots show the difference between estimated epigenetic age and reported chronologic age (linear regression residuals) for neonates with and without prenatal exposure to gestational diabetes. Lines within boxes denote medians, tops of boxes denote 75th percentiles, bottoms of boxes denote 25th percentiles, black vertical lines denote complete ranges, and black dots denote outliers.

**Figure 2.**
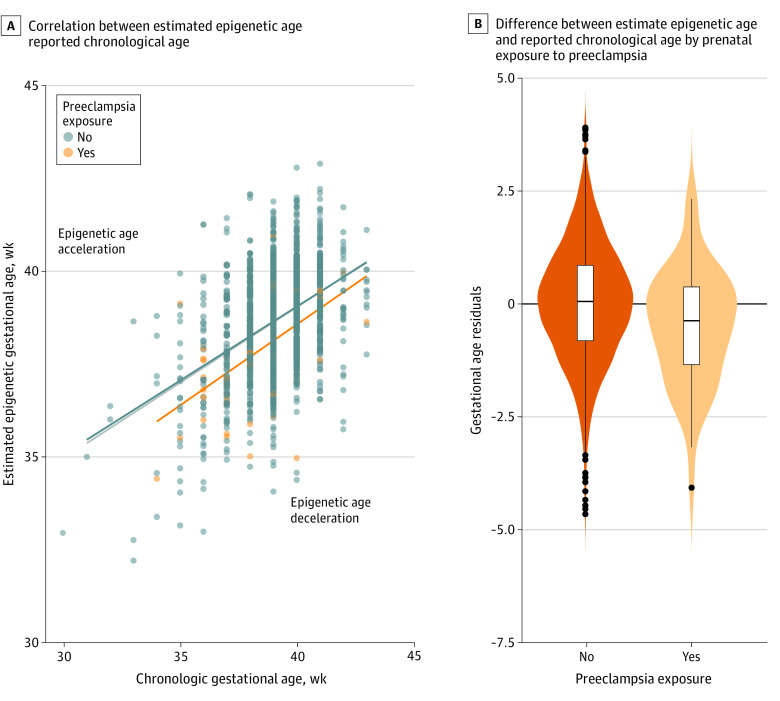
Association of Prenatal Exposure to Preeclampsia With Decelerated Epigenetic Gestational Age in Neonates A, Graph shows correlation between associated epigenetic age and reported chronological age. Each data point represents a participant’s gestational age at birth among neonates with and without exposure to preeclampsia during development. Lines represent the average residual values among a group of participants with or without exposure and in the overall sample (dark blue line). B, Violin plots show the difference between estimated epigenetic age and reported chronological age (linear regression residuals) for neonates with and without prenatal exposure to preeclampsia. Lines within boxes denote medians, tops of boxes denote 75th percentiles, bottoms of boxes denote 25th percentiles, black vertical lines denote complete ranges, and black dots denote outliers.

**Figure 3.**
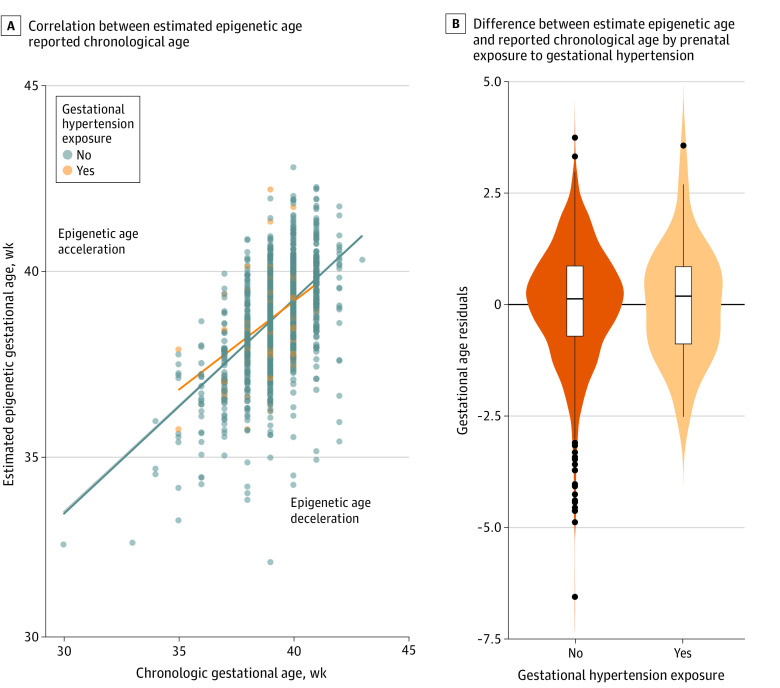
Association of Prenatal Exposure to Gestational Hypertension With Decelerated Epigenetic Gestational Age in Neonates A, Graph shows correlation between estimated epigenetic age and reported chronological age. Each data point represents a participant’s gestational age among neonates with and without exposure to gestational hypertension during development. Lines represent the average residual values among a group of participants with or without exposure and in the overall sample (dark blue line). B, Violin plots show the difference between predicted epigenetic age and reported chronologic age (linear regression residuals) for neonates with and without prenatal exposure to gestational hypertension. Lines within boxes denote medians, tops of boxes denote 75th percentiles, bottoms of boxes denote 25th percentiles, black vertical lines denote complete ranges, and black dots denote outliers.

Among the 1801 child participants included in our analyses, 1371 had information about the presence or absence of GD (89 [6%] with GD), 1039 had information about the presence or absence of GHT (57 [5%] with GHT), and 1458 had information about the presence or absence of PE (57 [4%] with PE). The frequencies of these pregnancy conditions are similar to expectations based on reports in US pregnant people. Mean maternal ages were comparable across exposure categories for each pregnancy complication with the exception of GD, where mothers with the condition were slightly older than those without. No significant differences in offspring sex or maternal education by pregnancy complication were observed. Mean prepregnancy BMI was higher among women with each of the pregnancy complications vs those without. In our main analyses, we observed that the presence of GD (β, −0.423; 95% CI, −0.709 to −0.138) or PE (β, −0.513; 95% CI, −0.857 to −0.170) was associated with lower offspring EAA and IAA at birth in both crude and adjusted models (Table 2, Figure 1, and Figure 2) vs neonates without GD or PE in utero exposure. Estimates were similar in magnitude for associations between GD or PE exposure with both EAA and IAA, suggesting the associations were not related to differences in blood cell type composition. The inclusion of covariates yielded little change in the magnitude of the main associations for either GD or PE (eTables 3 and 4 in Supplement 1). In contrast, we did not observe differences in EAA or IAA in offspring exposed to GHT vs those without exposure (β, 0.003; 95% CI, −0.338 to 0.344) (Table 2 and Figure 3).

**Table 2.** Multivariable Results for the Association Between Each Maternal Condition and Child Gestational Epigenetic Age Acceleration

Variable	Gestational diabetes (n = 1371)	Gestational hypertension (n = 1039)	Preeclampsia (n = 1458)
Extrinsic age acceleration^a^			
Crude			
β (95% CI)	−0.471 (−0.750 to −0.192)	0.007 (−0.334 to 0.349)	−0.519 (−0.860 to −0.178)
*P* value	.001	.97	.003
Adjusted^b^			
β (95% CI)	−0.423 (−0.709 to −0.138)	0.003 (−0.338 to 0.344)	−0.513 (−0.857 to −0.170)
*P* value	.004	.99	.003
Intrinsic age acceleration^c^			
Crude			
β (95% CI)	−0.434 (−0.705 to −0.162)	0.016 (−0.318 to 0.350)	−0.507 (−0.839 to −0.175)
*P* value	.002	.93	.003
Adjusted^b^			
β (95% CI)	−0.403 (−0.681 to −0.125)	0.006 (−0.327 to 0.340)	−0.517 (−0.850 to −0.183)
*P* value	.005	.97	.002

^a^ Extrinsic age acceleration measures are not adjusted for cord blood cell composition estimates.

^b^ Adjusted for maternal ethnicity, race, prepregnancy body mass index, age, prenatal smoking, educational level, and child sex.

^c^ Intrinsic age acceleration measures are adjusted for cord blood cell composition estimates.

Next, we evaluated whether the observed associations between GD, PE, or GHT and epigenetic age differed by child sex. Participant characteristics by sex are presented in eTables 5 and 6 in Supplement 1 and show similar trends as in the overall analytic sample. When we stratified the adjusted linear regression models by child sex, we found the associations between GD (β, −0.636; 95% CI, −1.070 to −0.200) and PE (β, −0.700; 95% CI, −1.189 to −0.210) and lower offspring EAA and IAA at birth were larger and statistically significant in female compared with male newborns (eTable 7 in Supplement 1). Association estimates for GHT were close to the null for both sexes.

## Discussion

In this cohort study that harmonized data from 12 pregnancy cohorts across the US, prenatal exposure to maternal GD and PE, but not to maternal GHT, was associated with lower biological maturity at birth vs maturity of unexposed neonates, an association mainly observed among female offspring. These findings are important because both conditions have been linked to short-term and long-term health impacts in the offspring.

Although a number of studies have examined associations of maternal prenatal psychosocial risk factors^38^ and diet^39^ with offspring GAA, only 2 studies^30,40^ have considered GD and hypertensive disorders of pregnancy, both from Finland. In contrast to our results, in the Prediction and Prevention of Preeclampsia and Intrauterine Growth Study,^30^ PE was positively associated with Knight GAA (ie, older biological gestational age at birth), with the largest associations for severe and early onset PE (1.37 weeks; 95% CI, 0.53 to 2.22 weeks). In that same study,^30^ deceleration of epigenetic gestational age was consistently observed among mothers treated with insulin for GD during a previous pregnancy (−1.44 weeks; 95% CI, −2.63 to −0.24 weeks). In contrast, investigators found no association between either maternal diabetes, a combination of prepregnancy diabetes and GD, or hypertensive disorders of pregnancy and cord blood epigenetic age in the Intrauterine Sampling in Early Pregnancy Study.^40^ However, in the latter study, investigators used the Bohlin gestational age clock, which differs also from our approach. Other studies have examined placenta epigenetic aging associations with prepregnancy conditions and birth outcomes in children. Similar to our findings, Tekola-Ayele et al^41^ reported sex-related differences in placenta epigenetic aging associations with fetal growth and birth outcomes in offspring. In contrast to our findings, the largest associations were observed in male offspring and not female offspring. This is not surprising given recent reports that epigenetic age deviations are not correlated and directions of epigenetic aging effects differ across tissues.^40^

When we stratified analyses by fetal sex, we observed associations for 2 pregnancy complications (GD and PE) with decelerated epigenetic aging among exposed vs unexposed female newborns but not among male newborns. To the extent that pregnancy complications represent physiological stressors, one interpretation of these findings is that female fetuses may respond to such stressors by slowing in utero maturation. However, male fetuses do not appear to similarly slow developmental maturation in response to these stressors. This interpretation is consistent with some prior evidence suggesting that female fetuses may be more responsive to the prenatal environment affecting growth than male fetuses. For example, in 24 000 infant-placenta pairs from the Collaborative Perinatal Project,^42^ placental chorionic plate size (a dimension that reflects the number of arteries available to supply blood) was more often associated with birth weight in female fetuses compared with male fetuses, suggesting greater modulation of growth in response to environmental cues among female fetuses.

Prior cohort studies have reported sex differences in GAA; however, in some cases, GAA was greater in male infants,^43^ whereas in others the results were the opposite.^30^ Still other studies reported no sex difference in GAA.^26,44^ Notably, to our knowledge, no prior studies have examined sex differences in the associations between pregnancy complications and GAA.

### Strengths and Limitations

A strength of our study is that it leveraged harmonized data from 12 cohorts across different regions of the US, providing much-needed representation of sociodemographic and geographic variability. Within this sampling complexity, GHT, GD, and PE rates were similar to those found in the US population. Another strength of the study is that it examined both extrinsic and intrinsic epigenetic age acceleration, which enables us to broadly compare the results of this study to prior works while controlling for biologically relevant covariates, such as cell composition, maternal BMI, and perinatal smoking.

This study also has limitations. Our multicohort harmonization approach introduced challenges, such as using 3 different arrays for epigenetic age determination. To create reliable estimates of epigenetic age in the context of these 3 arrays, we used the Knight clock. Although there are other epigenetic clocks developed for use in newborns,^25,45^ we chose to apply the clock that maximized inclusion of the greatest number of cohorts by including DNA methylation loci present across 3 types of arrays. Although our models included a robust set of harmonized covariates, we were limited in our ability to include a more extensive set of potential confounders, including folate intake during pregnancy, which could affect methyl donors available for genome DNA methylation processes.

Collection of our primary exposure variables leveraged both maternal self-report and medical record abstraction of GHT, GD, or PE. Several sources of information bias may be present in observational epidemiology studies such as ours, including exposure misclassification, missing not at random, recall bias, and survivor bias. Although it is possible our data may include some exposure misclassification, particularly for self-reported data, the majority of our exposure data was obtained from medical records, and exposure report is unlikely to be correlated with empirical epigenetic age measures. Thus, it is unlikely to have a major impact on our results. Recall bias is also unlikely given that the outcome in this study was an empirical biomarker measure collected at birth and unknown to parents who may have later reported their pregnancy conditions (a minority of our data was self-reported). We also guarded against missing not at random biases in our imputation procedure, as detailed in the Methods section. Survivor bias is possible in this study that had DNA methylation measures from live births only. To our knowledge, no studies have examined epigenetic aging among still birth or miscarriage outcomes; thus, it is unclear how lack of their inclusion here may influence the associations between PE, GD, or GHT and epigenetic aging in this study.

Given that the focus of this project was gestational onset for diabetes and hypertension, we did not investigate the associations of preexisting chronic hypertension and diabetes with epigenetic age acceleration or deceleration. Future studies are needed to determine whether the association we observed for GD and GHT are similar for neonates exposed to chronic diabetes during development. In addition, because of data mapping and harmonization protocols, chronological gestational age was collapsed to the week level to maximize sample size and inclusion of cohorts, which added imprecision to our outcome measure and potentially biased our results toward the null. Although we had a relatively large sample size for studies of this kind, pregnancy complications were rare (4%-6%); thus, our power to detect associations may have been limited. In addition, we were unable to assess the associations between the time of onset for each pregnancy condition and epigenetic age in newborns. It is possible that neonates with a longer period of exposure (ie, earlier pregnancy condition onset) could have had a more pronounced changes in the epigenetic aging pathway compared with those exposed later in pregnancy. Future studies are needed to evaluate exposure timing and dose-response patterns.

## Conclusions

In this cohort study, we leveraged extensive population resources from the ECHO program to advance our understanding of the associations of GD, PE, and GHT with developmental epigenetic aging processes in a diverse sample of mother-child dyads from across the US. We observed that prenatal exposure to GD and PE, but not to GHT, was associated with reduced biological maturity at birth among female neonates. These findings suggest that it will be important to further understand epigenetic mechanisms potentially at play in fetal programming and the relevance of the intrauterine environment on downstream health outcomes in offspring.

## Notes

Supplement 1. **eTable 1.** Description of Each of the 12 Participating ECHO Cohorts **eTable 2.** Descriptive Characteristics of Overall Population Sample and Our Final Analytic Sample (n=1,801) **eTable 3.** Multivariable Results for the Association Between Each Maternal Condition and Extrinsic Age Acceleration (EAA) in Child Cord Blood, When Varying Covariates in the Statistical Model **eTable 4.** Multivariable Results for the Association Between Each Maternal Condition and Intrinsic Gestational Accelerated Age (IAA) in the Child, at Birth, When Varying Covariates in the Statistical Model **eTable 5.** Descriptive Statistics Among 880 Male ECHO Participants in Our Sex-Stratified Analytic Sample **eTable 6.** Descriptive Statistics Among 921 Female ECHO Participants in Our Sex-Stratified Analytic Sample **eTable 7.** Sex-Stratified Multivariate Results for the Association Between Each Maternal Pregnancy Condition and Child Gestational Age Acceleration **eFigure.** Flowchart Describing the Number of ECHO Cohorts and Participants That Met the Inclusion Criteria for Our Analysis

Supplement 2.
Environmental Influences on Child Health Outcomes Program Collaborators

Supplement 3.
Data Sharing Statement
