# Supplementary material for: Analysis of Pregnancy Complications and Epigenetic Gestational Age of Newborns
Source: JAMA Netw Open. 2023 Feb 24;6(2):e230672. doi: 10.1001/jamanetworkopen.2023.0672 (PMC9958528; doi:10.1001/jamanetworkopen.2023.0672)
Supplement: Supplement 1. — eTable 1. Description of Each of the 12 Participating ECHO Cohorts eTable 2. Descriptive Characteristics of Overall Population Sample and Our Final Analytic Sample (n=1,801) eTable 3. Multivariable Results for the Association Between Each Maternal Condition and Extrinsic Age Acceleration (EAA) in Child Cord Blood, When Varying Covariates in the Statistical Model eTable 4. Multivariable Results for the Association Between Each Maternal Condition and Intrinsic Gestational Accelerated Age (IAA) in the Child, at Birth, When Varying Covariates in the Statistical Model eTable 5. Descriptive Statistics Among 880 Male ECHO Participants in Our Sex-Stratified Analytic Sample eTable 6. Descriptive Statistics Among 921 Female ECHO Participants in Our Sex-Stratified Analytic Sample eTable 7. Sex-Stratified Multivariate Results for the Association Between Each Maternal Pregnancy Condition and Child Gestational Age Acceleration eFigure. Flowchart Describing the Number of ECHO Cohorts and Participants That Met the Inclusion Criteria for Our Analysis [file jamanetwopen-e230672-s001.pdf]

Supplemental Online Content

Ladd-Acosta C, Vang E, Barrett ES, et al; Environmental Influences on Child Health Outcomes Program. Analysis of pregnancy complications and epigenetic gestational age of newborns. *JAMA Netw Open*. 2023;6(2):e230672.
doi:10.1001/jamanetworkopen.2023.0672

eTable 1. Description of Each of the 12 Participating ECHO Cohorts
eTable 2. Descriptive Characteristics of Overall Population Sample and Our Final Analytic Sample (n=1,801)
eTable 3. Multivariable Results for the Association Between Each Maternal Condition and Extrinsic Age Acceleration (EAA) in Child Cord Blood, When Varying Covariates in the Statistical Model
eTable 4. Multivariable Results for the Association Between Each Maternal Condition and Intrinsic Gestational Accelerated Age (IAA) in the Child, at Birth, When Varying Covariates in the Statistical Model
eTable 5. Descriptive Statistics Among 880 Male ECHO Participants in Our Sex-Stratified Analytic Sample
eTable 6. Descriptive Statistics Among 921 Female ECHO Participants in Our Sex-Stratified Analytic Sample
eTable 7. Sex-Stratified Multivariate Results for the Association Between Each Maternal Pregnancy Condition and Child Gestational Age Acceleration
eFigure. Flowchart Describing the Number of ECHO Cohorts and Participants That Met the Inclusion Criteria for Our Analysis

This supplemental material has been provided by the authors to give readers additional information about their work.

© 2023 Ladd-Acosta C et al. *JAMA Network Open.*

eTable 1. Description of Each of the 12 Participating ECHO Cohorts

Cohort	Recruitment methods and population characteristics	Enrollment year	DNAm: tissue; array	Sample size	Average gestational age (weeks)
Archive for Research in Child Health (ARCH)	Pregnant women recruited during their first prenatal visit at 1 of 3 prenatal clinics in Lansing, MI.	2008-2016	Blood spot; EPIC	Gestational Diabetes Mellitus: 5 cases and 132 controls<br>Gestational Hypertension: 0 cases and 0 controls<br>Preeclampsia: <5 cases and 13 controls	GDM Cases: 38.0<br>GDM Controls: 38.9<br><br>GHT Cases: -<br>GHT Controls: -<br><br>PE Cases: 39.0<br>PE Controls: 38.2
Columbia Center for Children's Environmental Health (CCCEH) and Mothers and Newborns (M and N)	Dominican or African American low-income mothers and their offspring recruited at OB/GYN clinics in New York, New York.	1998-2006	Cord blood; EPIC and 450K	Gestational Diabetes Mellitus: <5 cases and 0 controls<br>Gestational Hypertension: 0 cases and <5 controls<br>Preeclampsia: 6 cases and 134 controls	GDM Cases: 39.0<br>GDM Controls: -<br><br>GHT Cases: -<br>GHT Controls: 39.0<br><br>PE Cases: 40.0<br>PE Controls: 39.1
Conditions Affecting Neurocognitive Development and Learning in Early Childhood (CANDLE)	A majority African American sample of mother-child dyads recruited in pregnancy in Memphis, TN from safety net obstetrical clinics and local OB/GYN partners, television and radio advertisements, and directed mailings.	2006-2011	Cord blood; 27K	Gestational Diabetes Mellitus: <5 cases and 158 controls<br>Gestational Hypertension: 0 cases and <5 controls<br>Preeclampsia: 0 cases and 158 controls	GDM Cases: 40.0<br>GDM Controls: 39.0<br><br>GHT Cases: -<br>GHT Controls: 40.0<br><br>PE Cases: -<br>PE Controls: 39.0

© 2023 Ladd-Acosta C et al. *JAMA Network Open.*

Early Autism Risk Longitudinal Investigation (EARLI)	Pregnant women with a biological child with autism spectrum disorder (ASD) and the offspring of the current pregnancy, recruited from local community events, autism conferences, direct mail via autism service providers, and online and social media.	2009-2012	Cord blood; 450K	Gestational Diabetes Mellitus: <5 cases and 33 controls<br>Gestational Hypertension: 0 cases and 0 controls<br>Preeclampsia: 0 cases and 34 controls	GDM Cases: 39.0<br>GDM Controls: 38.9<br><br>GHT Cases: -<br>GHT Controls: -<br><br>PE Cases: -<br>PE Controls: 38.9
Fair Start	Dominican or African American low-income mothers and their offspring recruited at OB/GYN clinics in New York, NY.	2013-Current	Cord blood; EPIC	Gestational Diabetes Mellitus: <5 cases and 22 controls<br>Gestational Hypertension: 0 cases and <5 controls<br>Preeclampsia: <5 cases and 26 controls	GDM Cases: 40.0<br>GDM Controls: 39.6<br><br>GHT Cases: -<br>GHT Controls: 40.0<br><br>PE Cases: 40.0<br>PE Controls: 39.6
Healthy Start	Colorado community sample of mother-child dyads recruited in infancy from obstetrics clinics at a university hospital and by word of mouth, as well as medical university employees.	2009-2014	Cord blood; 450K	Gestational Diabetes Mellitus: 32 cases and 537 controls<br>Gestational Hypertension: 36 cases and 551 controls<br>Preeclampsia: 25 cases and 564 controls	GDM Cases: 38.8<br>GDM Controls: 39.1<br><br>GHT Cases: 38,9<br>GHT Controls: 39.1<br><br>PE Cases: 37.6<br>PE Controls: 39.2
Maternal and Developmental Risks from Environmental and Social Stressors (MADRES)	Pregnant women recruited from 3 OB/GYN clinics in Los Angeles, CA and their offspring.	2016-Current	Cord blood; EPIC	Gestational Diabetes Mellitus: 9 cases and 17 controls<br>Gestational Hypertension: <5 cases and 24 controls<br>Preeclampsia: <5 cases and 25 controls	GDM Cases: 38.7<br>GDM Controls: 39.2<br><br>GHT Cases: 39.5<br>GHT Controls: 39.0<br><br>PE Cases: 38.0<br>PE Controls: 39.1

© 2023 Ladd-Acosta C et al. *JAMA Network Open.*

New Hampshire Birth Cohort Study (NHBCS)	Pregnant women recruited from 5 prenatal clinics in the rural New Hampshire and their offspring.	2017-Current	Cord blood; 450K	Gestational Diabetes Mellitus: 8 cases and 123 controls<br>Gestational Hypertension: <5 cases and 128 controls<br>Preeclampsia: 0 cases and 131 controls	GDM Cases: 38.5<br>GDM Controls: 39.2<br><br>GHT Cases: 37.7<br>GHT Controls: 39.2<br><br>PE Cases: -<br>PE Controls: 39.1
Project Viva	Pregnant women receiving prenatal care at one of 8 urban and suburban clinics in and around Boston, MA and their offspring.	1999-2002	Cord blood; 450K	Gestational Diabetes Mellitus: 15 cases and 205 controls<br>Gestational Hypertension: 13 cases and 209 controls<br>Preeclampsia: 0 cases and 0 controls	GDM Cases: 39.1<br>GDM Controls: 39.6<br><br>GHT Cases: 38.8<br>GHT Controls: 39.2<br><br>PE Cases: -<br>PE Controls: -
Sibling Cohort	Dominican or African American low-income mothers and their offspring recruited at OB/GYN clinics in New York, NY.	2008-Current	Cord blood; 450K and EPIC	Gestational Diabetes Mellitus: <5 cases and 0 controls<br>Gestational Hypertension: 0 cases and <5 controls<br>Preeclampsia: 6 cases and 42 controls	GDM Cases: 38.5<br>GDM Controls: -<br><br>GHT Cases: -<br>GHT Controls: 38.5<br><br>PE Cases: 39.3<br>PE Controls: 38.8
The NYU Children's Health and Environment Study (NYU CHES)	Pregnant women recruited from prenatal clinics at NYU Langone Medical Center study sites.	2016-Current	Cord blood; EPIC	Gestational Diabetes Mellitus: 9 cases and 55 controls<br>Gestational Hypertension: <5 cases and 61 controls<br>Preeclampsia: <5 cases and 61 controls	GDM Cases: 38.1<br>GDM Controls: 38.9<br><br>GHT Cases: 37.3<br>GHT Controls: 38.9<br><br>PE Cases: 37.3<br>PE Controls: 38.9

© 2023 Ladd-Acosta C et al. *JAMA Network Open.*

eTable 2. Descriptive Characteristics of Overall Population Sample and Our Final Analytic Sample

	Missing Pregnancy Condition Data		Overall Participants
	No	Yes	
	(N=1,801)	(N=161)	(N=1,962)
**Chronological gestational age (weeks)**			
Median [Min, Max]	39.0 [30.0, 43.0]	39.0 [32.0, 43.0]	39.0 [30.0, 43.0]
**Maternal age (years)**			
Mean (SD)	28.6 (6.04)	29.3 (5.16)	28.7 (5.97)
**Child sex, N(%)**			
Male	880 (48.9%)	79 (49.1%)	959 (48.9%)
Female	921 (51.1%)	82 (50.9%)	1003 (51.1%)
**Ethnicity, N (%)**			
Non-Hispanic	1261 (70.0%)	120 (74.5%)	1,381 (70.4%)
Hispanic	524 (29.1%)	41 (25.5%)	565 (28.8%)
Missing	16 (0.9%)	0 (0%)	16 (0.8%)
**Race, N (%)**			
Asian	49 (2.7%)	<5	<55
Black	390 (21.7%)	27 (16.8%)	417 (21.3%)
Missing	244 (13.5%)	27 (16.8%)	271 (13.8%)
Other[a]	92 (5.1%)	<10	<105
White	1026 (57.0%)	94 (58.4%)	1120 (57.1%)
**Pre-pregnancy body mass index**			
Mean (SD)	26.2 (6.49)	27.7 (6.44)	26.3 (6.50)
Missing	128 (7.1%)	47 (29.2%)	175 (8.9%)
**Maternal educational level, N (%)**			
Less than high school	260 (14.4%)	17 (10.6%)	277 (14.1%)
High school degree, or equivalent	326 (18.1%)	20 (12.4%)	346 (17.6%)
Some college	364 (20.2%)	14 (8.7%)	378 (19.3%)
Bachelor's degree and above	629 (34.9%)	12 (7.5%)	641 (32.7%)
Missing	222 (12.3%)	98 (60.9%)	320 (16.3%)
**Prenatal Smoking, N (%)**			
No	1298 (72.1%)	49 (30.4%)	1347 (68.7%)
Yes	89 (4.9%)	51 (31.7%)	140 (7.1%)
Missing	414 (23.0%)	61 (37.9%)	475 (24.2%)

(n=1,801)

[a]Includes participants that reported any of the following: native hawaiian or other pacific islander, american indian or alaska native, multiple races, and other race not specified.

© 2023 Ladd-Acosta C et al. *JAMA Network Open.*

eTable 3. Multivariable Results for the Association Between Each Maternal Condition and Extrinsic Age Acceleration (EAA) in Child Cord Blood, When Varying Covariates in the Statistical Model

	**Gestational diabetes** (n=1371)	**Gestational hypertension** (n=1039)	**Preeclampsia** (n=1458)
**Model 1:** EAA ~ Pregnant condition			
β	-0.471	0.00747	-0.519
95% CI	(-0.75 , -0.192)	(-0.334 , 0.349)	(-0.86 , -0.178)
**Model 2:** EAA ~ Pregnant condition+ethnicity			
β	-0.482	0.0111	-0.527
95% CI	(-0.762 , -0.202)	(-0.331 , 0.353)	(-0.87 , -0.185)
**Model 3:** EAA ~ Pregnant condition+ethnicity+race			
β	-0.442	0.00722	-0.539
95% CI	(-0.724 , -0.16)	(-0.334 , 0.348)	(-0.881 , -0.198)
**Model 4:** EAA ~ Pregnant condition+ethnicity+race+BMI			
β	-0.439	0.0248	-0.503
95% CI	(-0.724 , -0.154)	(-0.318 , 0.367)	(-0.847 , -0.159)
**Model 5:** EAA ~ Pregnant condition+ethnicity+race+BMI+education			
β	-0.439	0.0157	-0.505
95% CI	(-0.724 , -0.154)	(-0.327 , 0.358)	(-0.85 , -0.161)
**Model 6: E**AA ~ Pregnant condition+ethnicity+race+mat BMI+education+mat age			
β	-0.436	0.0155	-0.502
95% CI	(-0.723 , -0.149)	(-0.327 , 0.358)	(-0.847 , -0.157)
**Model 7:** EAA ~ Pregnant condition+ethnicity+race+mat BMI+education+mat age+ child sex			
β	-0.42	0.0154	-0.502
95% CI	(-0.705 , -0.134)	(-0.325 , 0.356)	(-0.845 , -0.159)
**Model 8:** EAA ~ Pregnant condition+ethnicity+race+mat BMI+education+mat age +child sex+smoking			
β	-0.423	0.00299	-0.513
95% CI	(-0.709 , -0.138)	(-0.338 , 0.344)	(-0.857 , -0.17)
**Model 9:** EAA ~ Pregnant condition+ethnicity+race+mat BMI+education+mat age +child sex+smoking+CohortID			
β	-0.402	0.0084	-0.514
95% CI	(-0.688 , -0.116)	(-0.349, 0.332)	(-0.857, -0.17)

© 2023 Ladd-Acosta C et al. *JAMA Network Open.*

eTable 4. Multivariable Results for the Association Between Each Maternal Condition and Intrinsic Gestational Accelerated Age (IAA) in the Child, at Birth, When Varying Covariates in the Statistical Model

	**Gestational diabetes** (n=1371)	**Gestational hypertension** (n=1039)	**Preeclampsia** (n=1458)
**Model 1:** IAA ~ Pregnant condition			
β	-0.434	0.016	-0.507
95% CI	(-0.705 , -0.162)	(-0.318 , 0.35)	(-0.839 , -0.175)
**Model 2:** IAA ~ Pregnant condition+ethnicity			
β	-0.454	0.024	-0.522
95% CI	(-0.726 , -0.181)	(-0.31 , 0.358)	(-0.855 , -0.189)
**Model 3:** IAA ~ Pregnant condition+ethnicity+race			
β	-0.411	0.0193	-0.536
95% CI	(-0.685 , -0.136)	(-0.314 , 0.352)	(-0.868 , -0.205)
**Model 4:** IAA ~ Pregnant condition+ethnicity+race+BMI			
β	-0.415	0.0282	-0.508
95% CI	(-0.692 , -0.137)	(-0.306 , 0.363)	(-0.842 , -0.173)
**Model 5:** IAA ~ Pregnant condition+ethnicity+race+BMI+education			
β	-0.416	0.0195	-0.509
95% CI	(-0.693 , -0.138)	(-0.315 , 0.354)	(-0.844 , -0.175)
**Model 6: I**AA ~ Pregnant condition+ethnicity+race+mat BMI+education+mat age			
β	-0.414	0.0194	-0.507
95% CI	(-0.694 , -0.135)	(-0.315 , 0.354)	(-0.842 , -0.172)
**Model 7:** IAA ~ Pregnant condition+ethnicity+race+mat BMI+education+mat age+ child sex			
β	-0.4	0.0193	-0.507
95% CI	(-0.678 , -0.122)	(-0.313 , 0.352)	(-0.84 , -0.174)
**Model 8:** IAA ~ Pregnant condition+ethnicity+race+mat BMI+education+mat age +child sex+smoking			
β	-0.403	0.00659	-0.517
95% CI	(-0.681 , -0.125)	(-0.327 , 0.34)	(-0.85 , -0.183)
**Model 9:** EAA ~ Pregnant condition+ethnicity+race+mat BMI+education+mat age +child sex+smoking+CohortID			
β	-0.367	0.0134	-0.517
95% CI	(-0.645, -0.089)	(-0.345, 0.318)	(-0.85, -0.183)

© 2023 Ladd-Acosta C et al. *JAMA Network Open.*

eTable 5. Descriptive Statistics Among 880 Male ECHO Participants in Our Sex-Stratified Analytic Sample

	Gestational diabetes mellitus			Gestational hypertension			Preeclampsia		
	No (n=639)	Yes (n=51)	*P*	No (n=485)	Yes (n=29)	*P*	No (n=680)	Yes (n=28)	*P*
**Chronologic gestational age (weeks)**									
Median [min, max]	39.0 [30.0, 43.0]	39.0 [34.0, 41.0]	0.20	39.0 [30.0, 43.0]	39.0 [35.0, 41.0]	0.08	39.0 [32.0, 43.0]	38.5 [35.0, 43.0]	<0.05
**Maternal Age (years)**									
Mean (Standard deviation)	29.5 (5.80)	31.7 (6.04)	<0.05	30.0 (5.90)	31.1 (6.51)	0.38	28.3 (5.89)	29.4 (6.02)	0.34
**Ethnicity, N (%)**									
Non-Hispanic	520 (81.4)	36 (70.6)	0.10	381 (78.6)	26 (89.7)	0.30	461 (67.8)	13 (46.4)	<0.05
Hispanic	111 (17.4)	<15		96 (19.8)	3 (10.3)		211 (31.0)	15 (53.6)	
Missing	8 (1.3)	<5		8 (1.6)	0 (0)		8 (1.2)	0 (0)	
**Race, N (%)**									
Asian	18 (2.8)	7 (13.7)		19 (3.9)	0 (0)		17 (2.5)	<5	
Black	88 (13.8)	6(11.8)		51 (10.5)	<5		154 (22.6)	<5	
Missing	13 (2.0)	<10		10 (2.1)	0 (0)		98 (14.4)	9 (32.1)	
Other[a]	36 (5.6)	<5		25 (5.2)	<5		39 (5.7)	<5	
White	484 (75.7)	30 (58.8)	<0.01	380 (78.4)	25 (86.2)	0.69	372 (54.7)	15 (53.6)	0.21
**Pre-pregnancy body mass index (BMI)**									
Mean (Standard deviation)	26.3 (6.74)	31.2 (8.20)	<0.01	25.9 (6.48)	28.8 (8.18)	0.07	26.3 (6.63)	32.5 (9.76)	<0.01
Missing, N (%)	34 (5.3)	5 (9.8)		30 (6.2)	0 (0)		58 (8.5)	<5	
**Maternal educational, N (%)**									
Less than high school (HS)	47 (7.4)	<10	0.94	38 (7.8)	<5	0.82	93 (13.7)	7 (25.0)	0.59
HS degree or equivalent	74 (11.6)	6 (11.8)		65 (13.4)	<5		135 (19.9)	5 (17.9)	
Some college	136 (21.3)	13 (25.5)		108 (22.3)	5 (17.2)		135 (19.9)	6 (21.4)	
Bachelor's degree and above	284 (44.4)	23 (45.1)		259 (53.4)	18 (62.1)		217 (31.9)	9 (32.1)	
Missing	98 (15.3)	<5		15 (3.1)	<5		100 (14.7)	<5	
**Prenatal Smoking, N (%)**									
No	426 (66.7)	39 (76.5)	0.75	426 (87.8)	>24	0.27	506 (74.4)	26 (92.9)	0.25
Yes	35 (5.5)	<5		34 (7.0)	0 (0)		45 (6.6)	0 (0)	
Missing	178 (27.9)	>7		25 (5.2)	<5		129 (19.0)	<5	

[a]Includes participants that reported any of the following: native hawaiian or other pacific islander, american indian or alaska native, multiple races, and other race not specified.

© 2023 Ladd-Acosta C et al. *JAMA Network Open.*

eTable 6. Descriptive Statistics Among 921 Female ECHO Participants in Our Sex-Stratified Analytic Sample

	Gestational diabetes mellitus			Gestational hypertension			Preeclampsia		
	No (n=643)	Yes (n=38)	*P*	No (n=497)	Yes (n=28)	*P*	No (n=721)	Yes (n=29)	*P*
**Chronologic gestational age (weeks)**									
Median [min, max]	39.0 [33.0, 42.0]	39.0 [34.0, 42.0]	<0.05	39.0 [34.0, 42.0]	39.0 [35.0, 40.0]	0.06	39.0 [31.0, 43.0]	39.0 [34.0, 42.0]	<0.05
**Maternal Age (years)**									
Mean (SD)	29.3 (6.10)	31.3 (5.83)	<0.05	29.8 (6.26)	29.2 (6.93)	0.64	27.9 (5.98)	25.2 (6.18)	<0.05
**Ethnicity, N (%)**									
Non-Hispanic	525 (81.6)	23 (60.5)	<0.01	388 (78.1)	22 (78.6)	1	470 (65.2)	13 (44.8)	<0.05
Hispanic	112 (17.4)	>10		102 (20.5)	6 (21.4)		244 (33.8)	16 (55.2)	
Missing	6 (0.9)	<5		7 (1.4)	0 (0)		7 (1.0)	0 (0)	
**Race, N (%)**									
Asian	17 (2.6)	<5		18 (3.6)	<5		17 (2.4)	<5	
Black	118 (18.4)	5 (13.2)		64 (12.9)	7 (25.0)		189 (26.2)	7 (24.1)	
Missing	15 (2.3)	>5		9 (1.8)	0 (0)		128 (17.8)	9 (31.0)	
Other[a]	38 (5.9)	<5		31 (6.2)	<5		34 (4.7)	<5	
White	455 (70.8)	25 (65.8)	0.25	375 (75.5)	18 (64.3)	0.35	353 (49.0)	11 (37.9)	0.93
**Pre-pregnancy body mass index (BMI)**									
Mean (SD)	25.4 (5.98)	28.1 (6.50)	<0.05	25.0 (5.61)	26.3 (6.28)	0.30	26.1 (6.29)	27.1 (7.50)	0.52
Missing, N (%)	37 (5.8)	5 (13.2)		33 (6.6)	<5		63 (8.7)	5 (17.2)	
**Maternal educational, N (%)**									
Less than HS	65 (10.1)	<10	0.23	63 (12.7)	<5	0.50	137 (19.0)	6 (20.7)	0.08
HS degree or equivalent	82 (12.8)	9 (23.7)		70 (14.1)	7 (25.0)		144 (20.0)	7 (24.1)	
Some college	112 (17.4)	7 (18.4)		98 (19.7)	<10		130 (18.0)	10 (34.5)	
Bachelor's degree and above	278 (43.2)	13 (34.2)		256 (51.5)	13 (46.4)		198 (27.5)	<5	
Missing	106 (16.5)	<5		10 (2.0)	0 (0)		112 (15.5)	<5	
**Prenatal smoking, N (%)**									
No	445 (69.2)	26 (68.4)	0.92	442 (88.9)	25 (89.3)	0.40	532 (73.8)	23 (79.3)	0.88
Yes	29 (4.5)	<5		29 (5.8)	<5		41 (5.7)	<5	
Missing	169 (26.3)	>10		26 (5.2)	<5		148 (20.5)	<10	

HS= high school; SD = standard deviation

© 2023 Ladd-Acosta C et al. *JAMA Network Open.*

[a]Includes participants that reported any of the following: native hawaiian or other pacific islander, american indian or alaska native, multiple races, and other race not specified.

eTable 7. Sex-Stratified Multivariate Results for the Association Between Each Maternal Pregnancy Condition and Child Gestational Age Acceleration

	Gestational diabetes		Gestational hypertension		Preeclampsia	
**Child sex:**	**Male**	**Female**	**Male**	**Female**	**Male**	**Female**
**Extrinsic Age Acceleration (EAA)[a]**						
Crude:						
Beta coefficient (95% CI)	-0.309 (-0.674, 0.057)	-0.636 (-1.063, -0.21)	-0.059 (-0.531, 0.413)	0.0854 (-0.405, 0.575)	-0.363 (-0.841,0.115)	-0.666 (-1.148, -0.184)
P-value	0.10	0.003	0.81	0.73	0.14	0.01
Adjusted[b]:						
Beta coefficient (95% CI)	-0.251 (-0.632, 0.131)	-0.636 (-1.071, -0.2)	-0.034 (-0.513, 0.445)	0.1050 (-0.386, 0.596)	-0.325 (-0.814, 0.164)	-0.700 (-1.189, -0.21)
P-value	0.20	0.004	0.89	0.67	0.19	0.01
**Intrinsic Age Acceleration (IAA)[c]**						
Crude:						
Beta coefficient (95% CI)	-0.193 (-0.55, 0.164)	-0.706 (-1.12, -0.292)	-0.004 (-0.467, 0.46)	0.0449 (-0.432, 0.522)	-0.361 (-0.825, 0.103)	-0.645 (-1.114, -0.176)
P-value	0.29	0.001	0.99	0.85	0.13	0.01
Adjusted[b]:						
Beta coefficient (95% CI)	-0.133 (-0.505, 0.239)	-0.735 (-1.159, -0.312)	0.013 (-0.457, 0.484)	0.0601 (-0.419, 0.539)	-0.354 (-0.828, 0.121)	-0.678 (-1.154, -0.201)
P-value	0.48	0.001	0.96	0.81	0.14	0.01

[a]Extrinsic age acceleration (EAA) measures are not adjusted for cord blood cell composition estimates
[b]Adjusted for maternal ethnicity, race, pre-pregnancy body mass index, age, educational level, and prenatal smoking
[c]Intrinsic age acceleration (IAA) measures are adjusted for cord blood cell composition estimates

© 2023 Ladd-Acosta C et al. *JAMA Network Open.*

eFigure. Flowchart Describing the Number of ECHO Cohorts and Participants That Met the Inclusion Criteria for Our Analysis

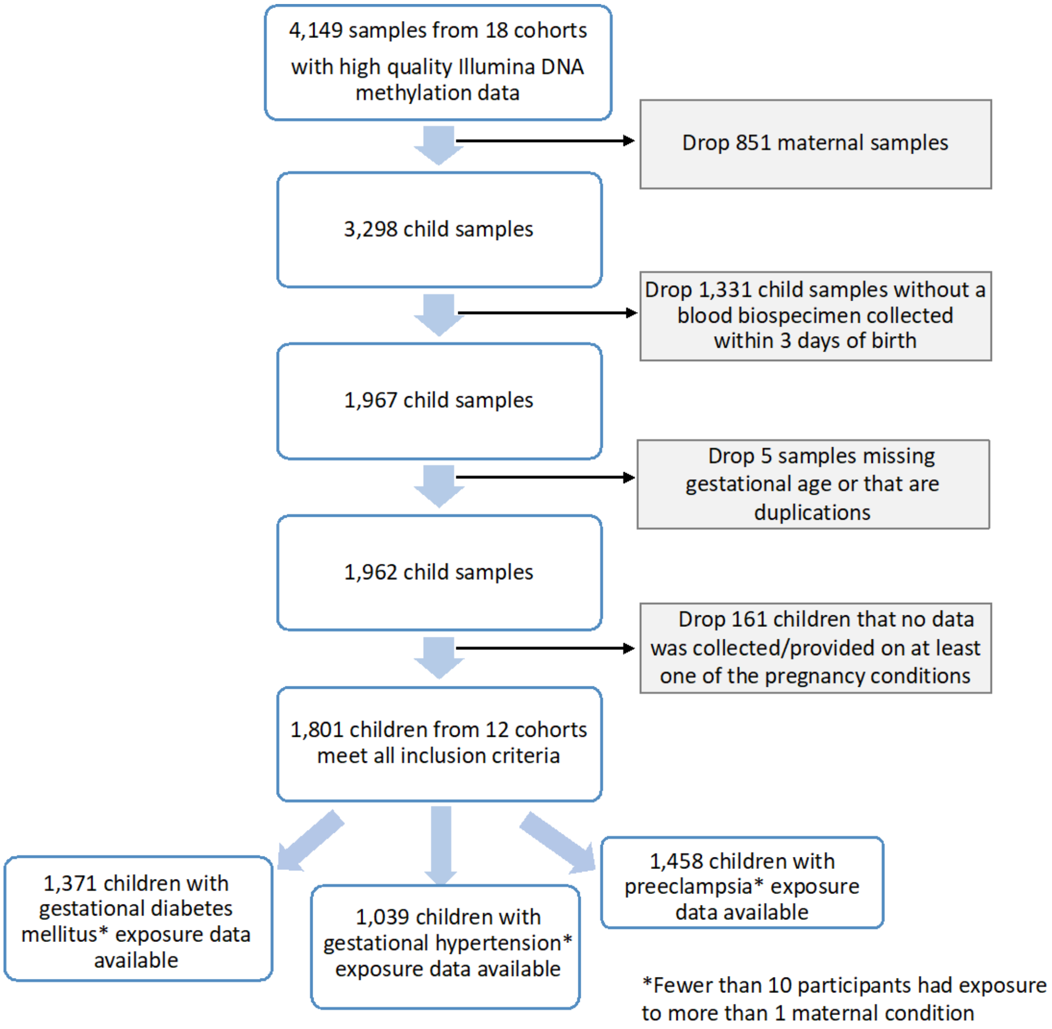

© 2023 Ladd-Acosta C et al. *JAMA Network Open.*
