# Supplementary material for: Analysis of Pregnancy Complications and Epigenetic Gestational Age of Newborns
Source: JAMA Netw Open. 2023 Feb 24;6(2):e230672. doi: 10.1001/jamanetworkopen.2023.0672 (PMC9958528; doi:10.1001/jamanetworkopen.2023.0672)
Supplement: Supplement 2. — Environmental Influences on Child Health Outcomes Program Collaborators [file jamanetwopen-e230672-s002.pdf]

Supplemental Online Content: Nonauthor Collaborators
*First name, last name, and suffix (if applicable) are required and will appear in PubMed.

*Group Name(s): Environmental Influences on Child Health Outcomes Program							
***First Name and Middle Initial(s)**	***Last Name**	***Suffix (eg, Jr, III)**	Academic Degrees	Institution	Location (city, state/province, country)	Role or Contribution, eg, chair, principal investigator	Group (if more than 1 Group listed in the byline) and/or Subgroup (eg, Steering Committee)
P B	Smith		MD, MHS, MPH	Coordinating Center: Duke Clinical Research Institute	Durham, North Carolina, USA	Principal investigator; Coordinating Center	
K L	Newby		MD	Coordinating Center: Duke Clinical Research Institute	Durham, North Carolina, USA	Principal investigator; Coordinating Center	
Lisa P	Jacobson		ScD	Data Analysis Center: Johns Hopkins University	Baltimore, Maryland, USA	Principal investigator; ECHO Data Analysis Center	
C B	Parker		PhD, MSPH	Data Analysis Center: Research Triangle Park Institute	Durham, North Carolina, USA	Principal investigator; ECHO Data Analysis Center	
Richard C	Gershon		PhD	Person-Reported Outcomes Core: Northwestern University	Evanston, Illinois, USA	Site PI	
David	Cella		PhD	Person-Reported Outcomes Core: Northwestern University	Evanston, Illinois, USA	PI; ECHO Pro-Core	
Theresa	Bastain		PhD, MPH	University of Southern California	Los Angeles, California, USA	Cohort PI	
Shohreh F	Farzan		PhD	University of Southern California	Los Angeles, California, USA	Cohort PI	
Rima	Habre		ScD, MSc	University of Southern California	Los Angeles, California, USA	Cohort PI	
Catherine	Karr		PhD, MD, MS	University of Washington: Department of Environmental and Occupational Health Sciences	Seattle, Washington, USA	Cohort PI	
Alex	Mason		PhD	University of Tennessee Health Sciences Center	Memphis, Tennessee, USA	Cohort PI	
Cindy T	McEvoy		MD, MCR	Oregon Health and Science University	Portland, Oregon, USA	Cohort PI	

Supplemental Online Content: Nonauthor Collaborators
*First name, last name, and suffix (if applicable) are required and will appear in PubMed.

***First Name and Middle Initial(s)**	***Last Name**	***Suffix (eg, Jr, III)**	Academic Degrees	Institution	Location (city, state/province, country)	Role or Contribution, eg, chair, principal investigator	Group (if more than 1 Group listed in the byline) and/or Subgroup (eg, Steering Committee)
Robert S	Tepper		MD, PhD	Indiana University, Riley Hospital for Children	Indianapolis, Indiana, USA	Cohort PI	
Lisa A	Croen		PhD	Kaiser Permanente Northern California Division of Research	Oakland, California, USA	Cohort PI	
Emily	Oken		MD, MPH	Project Viva: Harvard Pilgrim Health Care Institute	Boston, Massachusetts, USA	Cohort PI	
Jean	Kerver		PhD, MSc, MS	Michigan State University	East Lansing, Michigan, USA	Cohort PI	
Charles J	Barone		MD	Henry Ford Health System	Detroit, Michigan, USA	Cohort PI	
Patricia	McKane		DVM	Michigan Department of Health and Human Services	Lansing, Michigan, USA	Cohort PI	
Nigel	Paneth		MD, MPH	Michigan State University	East Lansing, Michigan, USA	Cohort PI	
Michael R	Elliott		PhD	University of Michigan	Ann Arbor, Michigan, USA	Cohort PI	
James	Gern		MD	University of Wisconsin	Madison, Wisconsin, USA	Cohort PI	
Russell S	Miller		MD	Columbia University Medical Center	New York, New York, USA	Cohort PI	
