# Supplementary material for: Analysis of Pregnancy Complications and Epigenetic Gestational Age of Newborns
Source: JAMA Netw Open. 2023 Feb 24;6(2):e230672. doi: 10.1001/jamanetworkopen.2023.0672 (PMC9958528; doi:10.1001/jamanetworkopen.2023.0672)
Supplement: Supplement 3. — Data Sharing Statement [file jamanetwopen-e230672-s003.pdf]

Data Sharing Statement

Ladd-Acosta. Analysis of Pregnancy Complications and Epigenetic Gestational Age of Newborns. *JAMA Netw Open*. Published February 24, 2023.
doi:10.1001/jamanetworkopen.2023.0672

Data
Data available: No

Additional Information
Explanation for why data not available: General data relevant to this publication is available through the ECHO portal at https://www.nih.gov/research-training/environmental-influences-child-health-outcomes-echo-program
